# Functional network analysis of gene-phenotype connectivity associated with temozolomide

**DOI:** 10.18632/oncotarget.20848

**Published:** 2017-09-12

**Authors:** Jia Shi, Bo Dong, Peng Zhou, Wei Guan, Ya Peng

**Affiliations:** ^1^ Department of Neurosurgery, The Third Affiliated Hospital of Soochow University, Changzhou City, 213003, China

**Keywords:** temozolomide, glioma, TP53

## Abstract

**Rationale:**

Glioma has a poor survival rate in patients even with aggressive treatment. Temozolomide (TMZ) is the standard chemotherapeutic choice for treating glioma, but TMZ treatment consistently leads to high resistance.

**Aim:**

To investigate the underlying mechanisms of TMZ action with new therapeutic regimens in glioma.

**Methods and results:**

The biological effects of TMZ mainly depend on the three following DNA repair systems: methylguanine methyltransferase (MGMT), mismatch repair (MMR) and base excision repair (BER). Based on related genes in these three systems, web-based tools containing data compiled from open-source databases, including DrugBank, STRING, WebGestalt and ClueGO, were queried, and five common genes along with the top fifteen pathways, including the glioma pathway, were identified. A genomic analysis of the six genes identified in the glioma pathway by cBioPortal indicated that TMZ might exert biological effects via interaction with the tumor protein P53(TP53) signaling axis. Finally, a survival analysis with the six genes in glioma cases (low-grade glioma and glioblastoma multiforme) was conducted using OncoLnc, which might provide directions for the future exploration of prognosis in glioma.

**Conclusions:**

This study indicates that a functional network analysis resembles a “BioGPS”, with the ability to draw a web-based scientific map that can productively and cost-effectively associate TMZ with its primary and secondary biological targets.

## INTRODUCTION

Glioma is the most common primary brain tumor in adults and has the highest degree of malignancy [1]. Currently, the standard therapy for glioma is maximal surgical resection followed by fractionated radiotherapy along with recurrent adjuvant treatment using alkylating agents such as nitrosoureas, procarbazine, and more recently, temozolomide (TMZ) [2, 3]. TMZ is a monofunctional cytostatic agent that has been synthesized at Aston University since 1984 [4]. TMZ has been reported as a clinical treatment for glioblastoma multiforme (GBM) and metastatic melanoma [5]. Recently, GBM has also been studied in refractory acute leukemia with promising results [6, 7].

The action of TMZ undergo DNA damage and DNA repair system two stages. In general, DNA repair systems are in charge of maintaining the genome integrity of cellular organism as they are able to neutralize DNA damage induced by various chemical and physical agents [8]. Therefore, for a better understanding of the molecular mechanisms of the biological effects of TMZ and target cellresistance to these agents it is necessary to consider underlying pathways of DNA repair. Like many other alkylating agents, the cytotoxic effects of TMZ is believed to manifest largely by the formation of methylation of the *O*6 position of guanine [9]. Consequently, the primary mechanisms of resistance to TMZ is a function of the activity of the DNA repair enzyme O(6)-alkylguanine-DNA-alkyltransferase, also called methylguanine methyltransferase (MGMT) [11]. During the process of DNA repair, MGMT also induces DNA mismatch repair system and base excision repair pathway [10, 11]. Thus, the biological effects of TMZ depend on at least three DNA repair systems [9]. (1) *MGMT* is a DNA repair protein encoded by the MGMT gene and is involved in the cellular defense against toxicity and mutagenesis from alkylating agents [12]. (2) The mismatch repair (MMR) system consists of a protein complex that contributes to the repair of biosynthetic errors generated during DNA replication. The MMR system recognizes and repairs not only base mismatches but also insertion and deletion loops [13]. (3) The base excision repair (BER) system, similar to the MMR system, is a protein complex that controls damage from cellular metabolic lesions (such as oxidation or the methylation of DNA bases) and from base modifications induced by physical or chemical agents [14].

Prior to the last decade, TMZ was shown to significantly improve outcomes in patients with GBM when administered concomitantly with radiotherapy and as maintenance strategy thereafter [15]. Nevertheless, despite intense therapy, the average survival rate of GBM patients is only 12-18 months [16, 17]. One critical barrier to the effective treatment of malignant glioma is resistance to TMZ, which has attracted significant scientific interest. As demonstrated previously, the biological effects of TMZ mainly depend on three DNA repair systems. However, the primary and secondary targets of TMZ and the interactions between the target genes of these three DNA repair systems remain unclear.

With the advancements in multicenter genomics studies, including microarrays, proteomics and high-throughput screening assays, exploratory network-based studies have been used to analyze massive amounts of data and numerous human diseases. In this study, we first employed DrugBank in a broad study of TMZ and drug-target information. Based on the three DNA repair systems, we identified five common targets using the STRING database. Then, the targets and associated genes were further explored through pathway enrichment analysis using the WebGestalt and ClueGO databases; the glioma pathway was screened, and related genes were identified to further explore genomic alterations using the cBio Cancer Genomics Portal (cBioPortal) database. Overall, our study based on these functional network analyses provides valuable information for exploring the underlying mechanisms of TMZ action against glioma and for identifying potential targets to overcome TMZ resistance in glioma.

## RESULTS

### Characterization of TMZ bioactivity using DrugBank and the visualization of TMZ linkage networks using STRING

**Figure 1:**
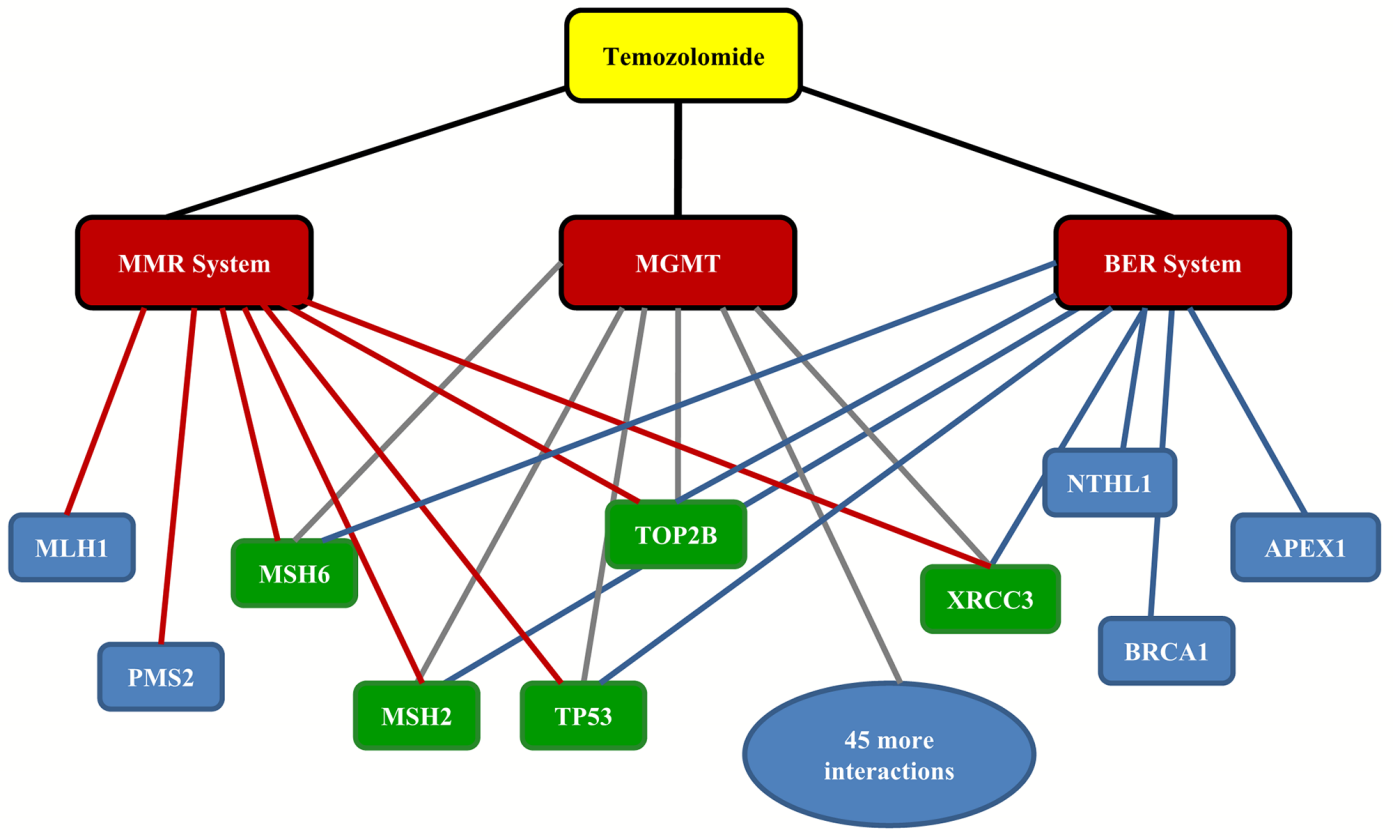
Drug-target protein interaction network of temozolomide Five protein targets (in green) in common among three DNA repair systems were identified using STRING : MSH2, MSH6, XRCC3, TOP2B and TP53. MGMT: methylguanine methyltransferase. MMR: mismatch repair. BER: base excision repair.

DrugBank was first queried using TMZ as an input, which resulted in the output DB00853 categorizing TMZ with alkylating agents, antineoplastic agents, imidazoles, immunosuppressive agents, noxae, toxic actions and triazenes (Table 1). Furthermore, grouping TMZ as a Food and Drug Administration (FDA)-approved drug, the indications of TMZ were divided into 3 detailed classes: (1) treatment of adult patients with anaplastic astrocytoma after nitrosourea and procarbazine therapy; (2) concomitant administration with radiation treatment for patients with newly diagnosed GBM; and (3) maintenance therapy for patients with GBM (Table 1). Table 2 illustrates that the direct target of TMZ is DNA (Table 2). As mentioned previously, the biological effects of TMZ primarily depend on three DNA repair systems: MGMT, MMR and BER. The MMR and BER systems consist of protein complexes; the MMR system includes the following 8 genes: MRC1, ATP9B, MSH6, MSH3, EXO1, MLH1, PMS2 and MSH2, while the BER system includes the following 14 genes: RAD1, RAD9A, HUS1, NTHL1, PARP1, PARP2, PARP3, PNKP, APEX1, POLL, TDG, FEN1, USP47 and APEX2 (Table 3). Next, we analyzed MGMT-related genes using STRING, and a total of 50 target protein interactions were identified (Supplementary Table 1). No gene common to all 3 DNA repair systems was detected. Therefore, we expanded our search and further analyzed MMR/BER-related protein-protein interactions (PPIs); we ultimately identified five genes in common (MSH2, MSH6, TOP2B, TP53 and XRCC3) that were related to the three DNA repair systems (Figure 1). It should be noted that all five of the common genes have been reported to be directly associated with glioma [18–22], suggesting potential roles of these genes in mediating the action of TMZ against glioma.

**Table 1:** Characterization of Temozolomide using DrugBank

DB_ID	Name	Group	Category	Indication
DB00853	Temozolomide	Approved	Alkylating AgentsAntineoplastic AgentsImidazolesImmunosuppressive Agents	1. For the treatment of adult patients diagnosed with anaplastic astrocytoma who has progressed after nitrosourea and procarbazine therapy.
			NoxaeToxic ActionsTriazenes	2. Concomitantly with radiation therapy for treatment of newly diagnosed glioblastoma multiforme.
				3. Used as maintenance therapy for glioblastoma multiforme.

**Table 2:** Identifcation of direct targets of Temozolomide using DrugBank

DB_ID	Name	Target	Kind	Pharmacological action	Action	Organism
DB00853	Temozolomide	DNA	Nucleotide	Yes	Cross-linking/Alkylation	Human

**Table 3:** Targets of Temozolomide by STRING

Searched_Drug(1/1)				
DB_ID	Name	Target	Gene	Entrez_ID
DB00853	Temozolomide	MGMT	MGMT	4255
DB00853	Temozolomide	Mismatch repair	MRC1	4360
			ATP9B	374868
			MSH2	4436
			MSH3	4437
			MSH6	2956
			EXO1	9156
			MLH1	4292
			PMS2	5395
DB00853	Temozolomide	Base excision repair	RAD1	5810
			RAD9A	5883
			HUS1	3364
			NTHL1	4913
			PARP1	142
			PARP2	10038
			PARP3	10039
			PNKP	11284
			POLL	27343
			TDG	6996
			FEN1	2237
			USP47	55031
			APEX1	328
			APEX2	27301

### Analysis of functional attributes associated with TMZ-mediated changes in gene sets using WebGestalt and ClueGO

To evaluate the functional features of TMZ-mediated gene sets, we performed a Kyoto Encyclopedia of Genes and Genomes (KEGG) pathway enrichment analysis using WebGestalt. The top 15 KEGG pathways were identified based on genes associated with the three DNA repair systems (Table 4). These pathways included BER (11 genes), platinum drug resistance (11 genes), pathway in cancer (19 genes), bladder cancer (8 genes), non-small cell lung cancer (8 genes), MMR (6 genes), endometrial cancer (7 genes), colorectal cancer (7 genes), central carbon metabolism in cancer (7 genes), melanoma (7 genes), breast cancer (8 genes), glioma (6 genes), microRNA (miRNA) in cancer (10 genes), prostate cancer (6 genes) and apoptosis (7 genes). All the identified KEGG enrichment pathways represented biological functions that had statistically significant associations with the TMZ gene sets, thus motivating further investigation. A broad grouping of the functional analysis results indicated that the TMZ-linked genes were mainly associated with (1) DNA repair and drug resistance pathways, (2) cancer-related signaling pathways, and (3) signaling pathway cascades with potential mechanistic underpinnings, including the control of cancer cell development and survival via miRNA-mediated carbon metabolism and the regulation of apoptosis.

**Table 4:** List of enriched Temozolomide associated gene sets identified using WebGestalt Analysis

Pathway Name	#Gene	Gene (corresponding gene set)	statistics
Base excision repair	11	PARP2;PARP3;PARP1;FEN1;APEX2;POLL;APEX1;NTHL1;OGG1;TDG;XRCC1	C=33; O=11; E=0.21;R=51.99; *p* value=0
Platinum drug resistance	11	CDKN2A;GSTP1;MSH6;BIRC5;MLH1;MSH2;MSH3;BRCA1;TOP2A;TOP2B;TP53	C=75; O=11; E=0.48;R=22.87; *p* value=7.61E-13
Pathway in cancer	19	CDKN2A;CDKN2B;RASSF1;DAPK1;EGFR;GSTP1;MSH6;HIF1A;HRAS;BIRC5;KRAS;MLH1;MSH2;MSH3;PTEN;RARB;BRCA2;TP53;CDH1	C=397; O=19; E=2.55;R=7.46; *p* value=8.94E-13
Bladder cancer	8	CDKN2A;RASSF1;DAPK1;EGFR;HRAS;KRAS;TP53;CDH1	C=41; O=8; E=0.26;R=30.43; *p* value=1.23E-10
Non-small cell lung cancer	8	CDKN2A;RASSF1;EGFR;FHIT;HRAS;KRAS;RARB;TP53	C=56; O=8; E=0.36;R=22.28; *p* value=1.70E-09
Mismatch repair	6	MSH6;MLH1;MSH2;MSH3;PMS2;EXO1	C=23; O=6; E=0.15;R=40.69; *p* value=4.61E-09
Endometrial cancer	7	EGFR;HRAS;KRAS;MLH1;PTEN;TP53;CDH1	C=52; O=7; E=0.33;R=21.00; *p* value=2.98E-08
Colorectal cancer	7	MSH6;BIRC5;KRAS;MLH1;MSH2;MSH3;TP53	C=62; O=7; E=0.40;R=17.61; *p* value=1.05E-07
Central carbon metabolism in cancer	7	EGFR;HIF1A;HRAS;IDH1;KRAS;PTEN;TP53	C=67; O=7; E=0.43;R=16.30; *p* value=1.80E-07
Melanoma	7	CDKN2A;EGFR;HRAS;KRAS;PTEN;TP53;CDH1	C=71; O=7; E=0.46;R=15.38; *p* value=2.71E-07
Breast cancer	8	EGFR;ESR1;HRAS;KRAS;PTEN;BRCA1;BRCA2;TP53	C=146; O=8; E=0.94;R=8.55; *p* value=3.29E-06
Glioma	6	CDKN2A;EGFR;HRAS;KRAS;PTEN;TP53	C=66; O=6; E=0.42;R=14.18; *p* value=3.38E-06
MicroRNAs in cancer	10	CDKN2A;RASSF1;DNMT1;EGFR;HRAS;KRAS;PTEN;BRCA1;TIMP3;TP53	C=299; O=10; E=1.92;R=5.22; *p* value=1.46E-05
Prostate cancer	6	EGFR;GSTP1;HRAS;KRAS;PTEN;TP53	C=89; O=6; E=0.57;R=10.51; *p* value=1.93E-05
Apoptosis	7	PARP2;PARP3;PARP1;HRAS;BIRC5;KRAS;TP53	C=140; O=7; E=0.90;R=7.80; *p* value=2.63E-05

The row lists the following statistics: C: the number of reference genes in category; O: the number of genes in the gene set and also in the category; E: the expected number in the category; R: ratio of enrichment; *p* value: *p* value from hypergeometric test.

**Figure 2:**
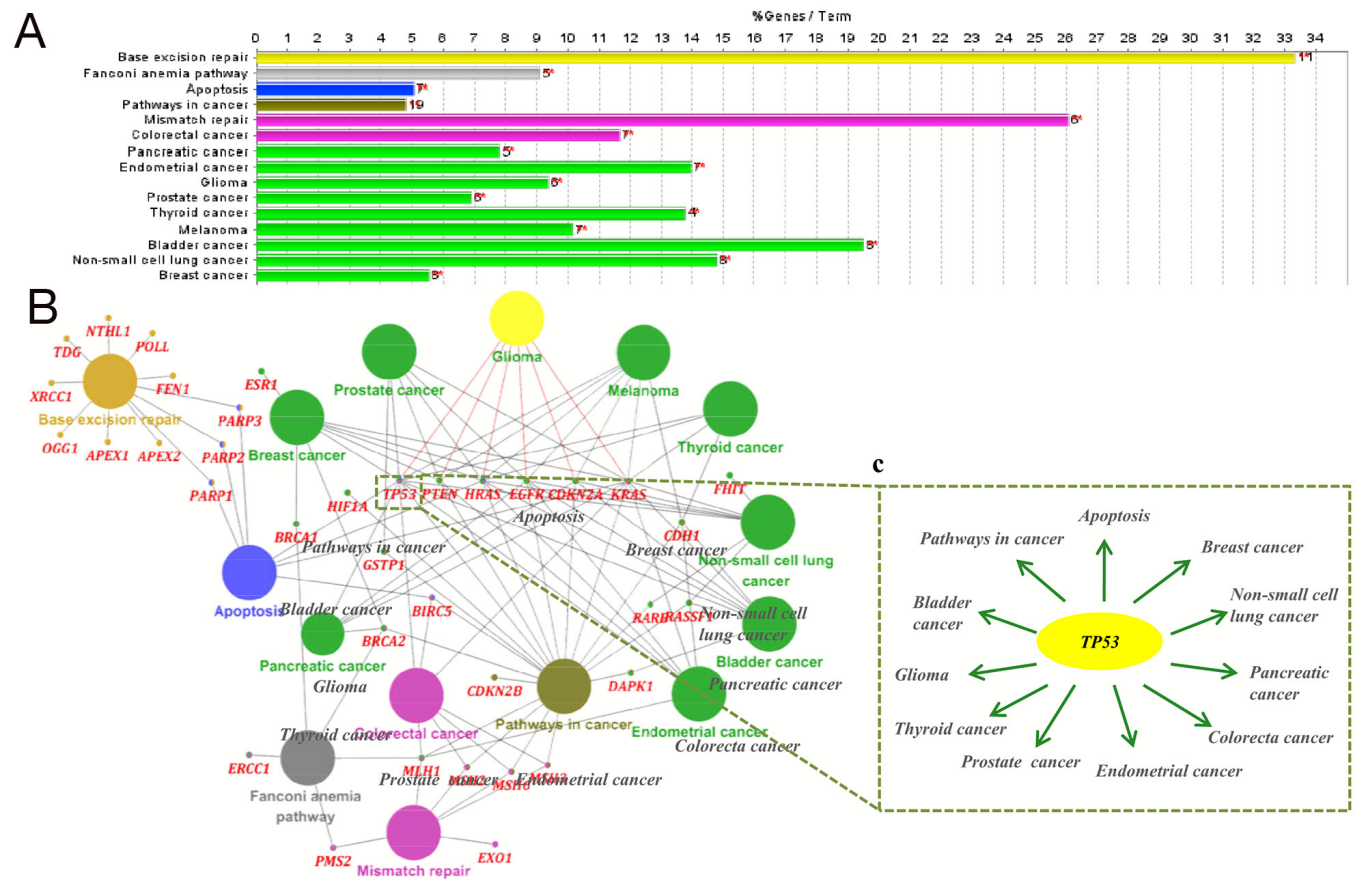
Kyoto Encyclopedia of Genes and Genomes (KEGG) pathway analysis of temozolomide -associated gene sets performed using ClueGO **(A)** The top 15 statistically enriched KEGG pathways and involved gene numbers (more details can be found in Table 5). **(B)** The biological network of temozolomide -linked genes consists of the top 15 statistically enriched KEGG pathways (large circle with different color), corresponding genes (red) and STRING protein interaction. **(C)** *TP53* (in yellow) is shown as an illustration of complex regulatory networks. Among the top 15 KEGG pathways, 11 were associated with TP53.

Next, to validate the pathways identified by WebGestalt, we additionally performed KEGG pathway enrichment using ClueGO. The glioma pathway was also screened for statistical significance in this analysis (Figure 2A and Table 5). A broad search revealed that 6 genes in the glioma pathway were connected to TMZ-linked genes: CDKN2A, EGFR, HRAS, KRAS, PTEN and TP53 (Figure 2B and Table 5). Notably, among these 6 selected genes (identified by both WebGestalt and ClueGO), TP53was found to participate in numerous signaling pathways, including apoptosis and several cancer-related pathways (Figure 2C), suggesting a critical role for TP53 in diverse biological effects mediated by TMZ.

**Table 5:** List of enriched Temozolomide associated gene sets identified using ClueGO Analysis

Pathway Name	#Gene	Gene (corresponding gene set)	*p* value
Base excision repair	11	APEX1, APEX2, FEN1, NTHL1, OGG1, PARP1, PARP2, PARP3, POLL, TDG, XRCC1	45.0E-18
Fanconi anemia pathway	5	BRCA1, BRCA2, ERCC1, MLH1, PMS2	26.0E-6
Apoptosis	7	BIRC5, HRAS, KRAS, PARP1, PARP2, PARP3, TP53	27.0E-6
Pathways in cancer	19	BIRC5, BRCA2, CDH1, CDKN2A, CDKN2B, DAPK1, EGFR, GSTP1, HIF1A, HRAS, KRAS, MLH1, MSH2, MSH3, MSH6, PTEN, RARB, RASSF1, TP53	1.2E-12
Mismatch repair	6	EXO1, MLH1, MSH2, MSH3, MSH6, PMS2	5.1E-9
Colorectal cancer	7	BIRC5, KRAS, MLH1, MSH2, MSH3, MSH6, TP53	95.0E-9
Pancreatic cancer	5	BRCA2, CDKN2A, EGFR, KRAS, TP53	54.0E-6
Endometrial cancer	7	CDH1, EGFR, HRAS, KRAS, MLH1, PTEN, TP53	25.0E-9
Glioma	6	CDKN2A, EGFR, HRAS, KRAS, PTEN, TP53	3.1E-6
Prostate cancer	6	EGFR, GSTP1, HRAS, KRAS, PTEN, TP53	18.0E-6
Thyroid cancer	4	CDH1, HRAS, KRAS, TP53	33.0E-6
Melanoma	7	CDH1, CDKN2A, EGFR, HRAS, KRAS, PTEN, TP53	250.0E-9
Bladder cancer	8	CDH1, CDKN2A, DAPK1, EGFR, HRAS, KRAS, RASSF1, TP53	140.0E-12
Non-small cell lung cancer	8	CDKN2A, EGFR, FHIT, HRAS, KRAS, RARB, RASSF1, TP53	1.4E-9
Breast cancer	8	BRCA1, BRCA2, EGFR, ESR1, HRAS, KRAS, PTEN, TP53	0.0000034

### Mining genomic alterations associated with TMZ-related genes in glioma using cBioPortal

**Figure 3:**
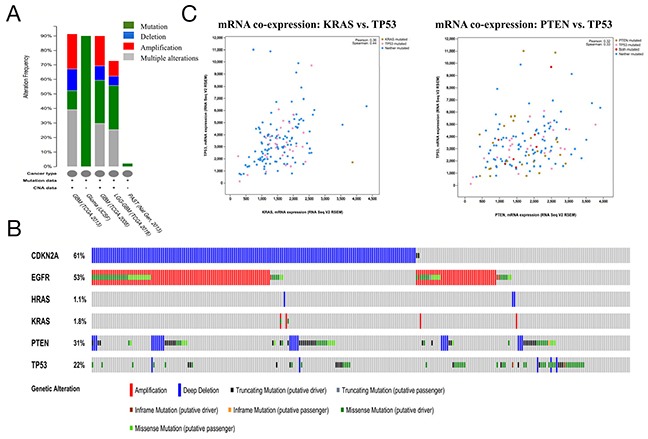
Mining genetic alterations connected with the temozolomide -associated genes CDKN2A, EGFR, HRAS, KRAS, PTEN and TP53 in glioma studies embedded in cBioPortal **(A)** Overview of changes in the CDKN2A, EGFR, HRAS, KRAS, PTEN and TP53 genes in the genomics datasets available in 5 different glioma studies. **(B)** Oncopoint: a visual summary of genomic alterations across a set of glioblastoma multiforme (GBM) samples (data taken from the Brennan CW study, Cell, 2013) based on a query of 6 genes (CDKN2A, EGFR, HRAS, KRAS, PTEN and TP53). Different genomic alterations involving mutations and CNAs (copy number alterations, gene amplifications and homozygous deletions) are summarized, color-coded and displayed as % change in specific affected genes in individual glioma samples. Each row represents a gene, and each column represents a sample. Red bars represent amplifications, blue bars represent homozygous deletions, and green bars represent nonsynonymous mutations. **(C)** mRNA co-expression of KRAS A. with TP 53 and PTEN with TP53

To further validate the relationship between TMZ-related genes and the glioma pathway, cBioPortal was used to explore genomic alterations in genes connected with TMZ treatment of glioma. A total of 8 glioma studies were included in cBioPortal [23–27]. Three studies were provisionally embraced; thus, emphasis was directed to the remaining 5 studies. A query of the 6 selected genes (CDKN2A, EGFR, HRAS, KRAS, PTEN and TP53) identified in the glioma pathway was performed, and these genes were analyzed in the 5 glioma studies. Alterations ranging from 2.1% to 91.5% were detected for submitted gene sets (Figure 3A). A summary of multiple gene alterations detected across each set of tumor samples from the study of Brennan CW showed the most pronounced genomic changes among the 5 glioma studies [26]. In this study, 257 cases (91.5%) had alterations in all 6 genes; the frequency of alterations in each gene is shown in Figure 3B. For CDKN2A (61%), most alterations were classified as deep deletions, with a few cases of truncating mutations. For EGFR (53%), the majority of alterations were amplifications, with a small fraction of missense mutations. For PTEN (31%) and TP53 (22%), the gene changes included deep deletions and truncating, missense and inframe mutations. For KRAS and HRAS, few alterations were detected with 1.8% and 1.1%, respectively (Figure 3B). An analysis of interactions between genes showed that KRAS and TP53 (Pearson's correlation: 0.36) as well as PTEN and TP53 (Pearson's correlation: 0.32) exhibited co-expression in the GBM samples in the study of Brennan CW (Table 6), while CDKN2A and TP53 (*p*-value<0.001) as well as EGFR and TP53 (*p*-value=0.001) exhibited mutual exclusivity (Table 7), suggesting a central axis function for TP53 under TMZ control in the glioma pathway.

**Table 6:** Co-expressions of selected 6 mRNAs (CDKN2A, EGFR, HRAS, KRAS, PTEN and TP53) in Brennan C.W. study

Gene	Correlated gene	Cytoband	Pearson's Correlation	Spearman's Correlation
KRAS	TP53	17p13.1	0.36	0.44
PTEN	TP53	17P13.1	0.32	0.33

**Table 7:** Mutual exclusivity of selected 6 mRNAs (CDKN2A, EGFR, HRAS, KRAS, PTEN and TP53) in Brennan CW study

Gene A	Gene B	*p* Value	Log Odds Ratio	Association
CDKN2A	TP53	<0.001	-1.353	Tendency towards mutual exclusivity
EGFR	TP53	0.001	-0.939	Tendency towards mutual exclusivity
CDKN2A	EGFR	0.006	0.647	Tendency towards co-occurrence

**Figure 4:**
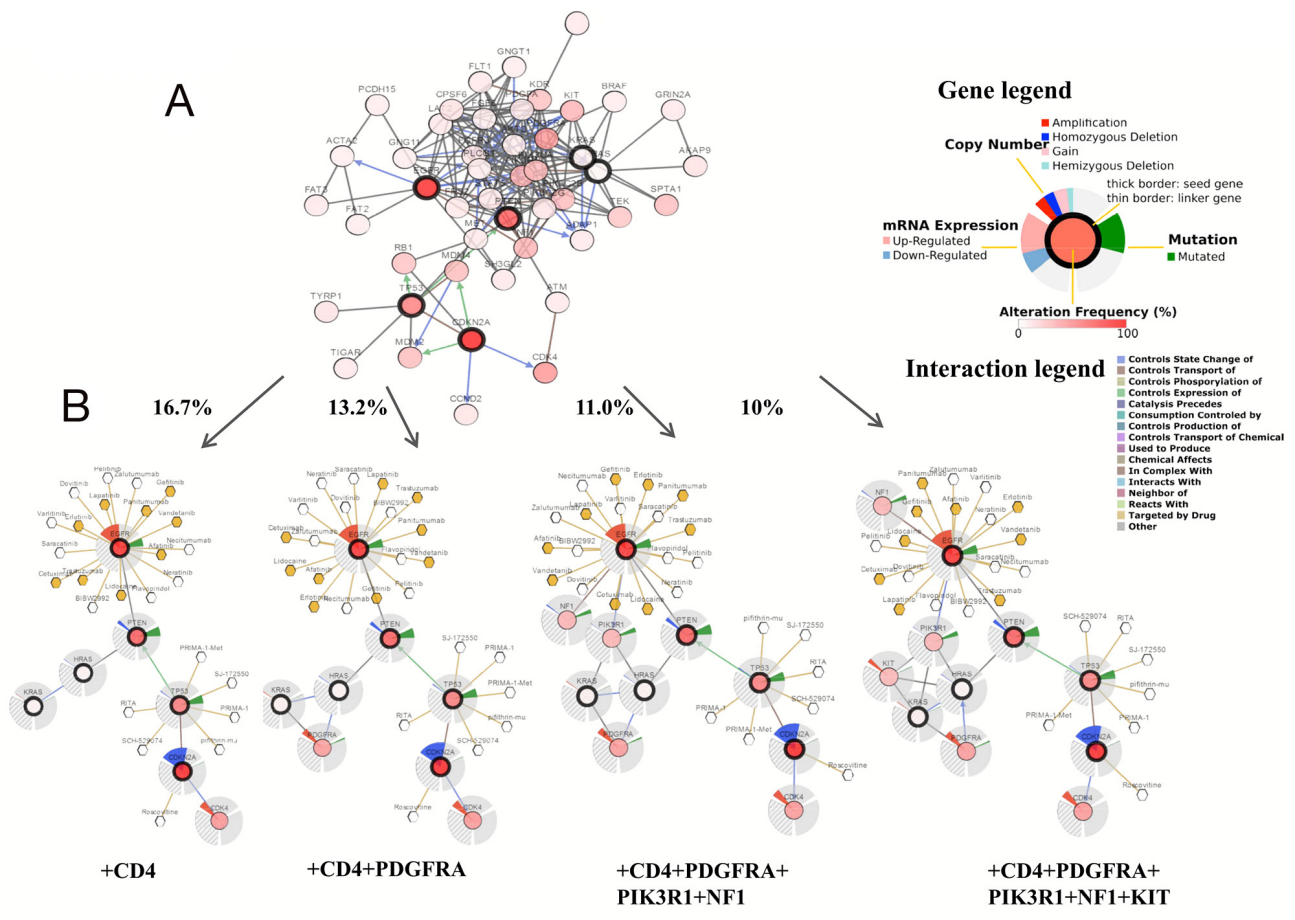
A visual display of gene networks connected to CDKN2A/EGFR/HRAS/KRAS/PTEN/TP53 in glioblastoma multiforme (GBM) (based on the study of Brennan CW, Cell, 2013) **(A)** Cross-cancer alteration summary for CDKN2A/EGFR/HRAS/KRAS/PTEN/TP53 mined from the cBioPortal for Cancer Genomics. Six selected genes and temozolomide-linked genes were applied as seed genes (indicated with thick black border) to automatically harvest all other genes identified as altered in GBM (data taken from the Brennan CW study, Cell, 2013). **(B)** Neighboring genes connected to the 6 query genes, filtered by alterations (%). Multidimensional genomic information and drug administration for a specific gene are exhibited for the seed genes (CDKN2A, EGFR, HRAS, KRAS, PTEN and TP53). Darker red indicates increased frequency of alteration (defined as a mutation, homozygous deletion and copy number amplification). The filters used involved the highest genomic alteration frequency within the selected GBM study in addition to the query genes.

We additionally used cBioPortal to perform interactive analysis and construct networks of genes that were altered in cancer. We created a network containing all neighbors of the 6 query genes (Figure 4A). To reduce the complexity of the analysis, we applied genomic alteration frequency within the selected glioma studies as a filter, such that only neighbors with a high alteration frequency are shown (Figure 4B). First, the 6 selected genes were determined to be associated with CD4 when a filter of ≥ 16.7% alteration was applied to neighbors. In comparison, 8 genes, including CD4 and PDGFRA, were evident using a filter of≥13.2% alteration. Ten gene clusters, including CD4, PDGFRA, NF1 and PIK3R1, were demonstrated when the filter was reduced to 11% alteration, while 11 genes, including CD4, PDGFRA, NF1, PIK3R1 and KIT, were revealed with a filter of 10% alteration. The comprehensive and pruned networks revealed the potential interactions as well as the complexity and the variability of differences in the interactions between TMZ-linked genes and the GBM samples in the Brennan CW study. Moreover, drugs that are applied for specific genes, including cancer drugs, FDA-approved drugs and others, are also shown in cBioPortal. Figure 4B lists specific cancer drugs acting on EGFR, TP53 and CDKN2A (Figure 4B);currently, TMZ is not used to target any of these genes. This network analysis provides a molecular basis for future clinical applications of TMZ targeting selected genes.

**Figure 5:**
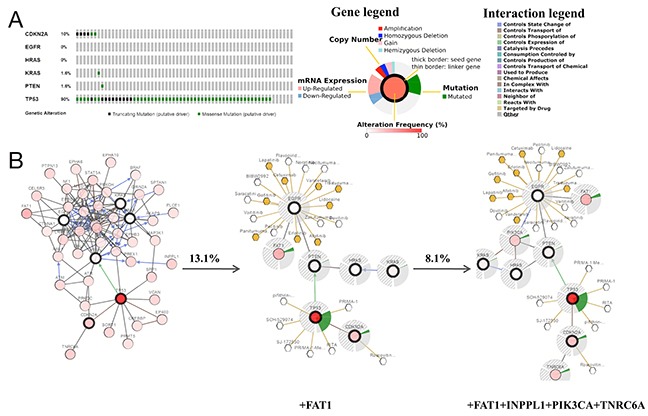
Genetic alterations and a visual display of the gene network connected to CDKN2A/EGFR/HRAS/KRAS/PTEN/TP53 in brain low-grade glioma (LGG) (based on the study of Johnson BE, Science 2014) **(A)** Oncopoint: a visual summary of genomic alterations across a set of LGG samples (data taken from Johnson BE, Science, 2014) based on a query of 6 genes (CDKN2A, EGFR, HRAS, KRAS, PTEN and TP53). **(B)** Neighboring genes connected to the6 query genes, filtered by alterations (%).

Low-grade gliomas (LGGs) in the brain are slower growing than their high-grade counterparts. LGGs account for 10-20% of all primary brain tumors, and the median survival of LGG patients ranges from 4.7 to 9.8 years [28]. Related literature revealed that TMZ is also indicated for LGG patients with high-risk features [29]. Figure 3A shows that among the LGG studies in cBioPortal, the study of Johnson BE observed the most pronounced genomic changes (Figure 3A, column 2) [2, 3]. In this study, gene sets were altered in 90.2% of 61 cases. *TP53* (90%) exhibited the most prominent changes, which were classified as missense mutations with a few cases of truncating mutations. For CDKN2A, alterations also included missense and truncating mutations. For EGFR, HRAS, KRAS and PTEN, few alterations existed in LGG cases (Figure 5A). Results from co-expression and mutual exclusivity analyses were not statistically significant (data not shown). Next, a network analysis was performed that included neighbors of the 6 query genes (Figure 5B). Seven genes, including FAT1, wererevealed using a filter of≥13.1% alteration;10 gene clusters (including FAT1, INPPL1, PIK3CA and TNRC6A) were shown when the filter was reduced to 8.1% alteration.

### Analysis of survival associated with TMZ-related genes in glioma according to OncoLnc

**Figure 6:**
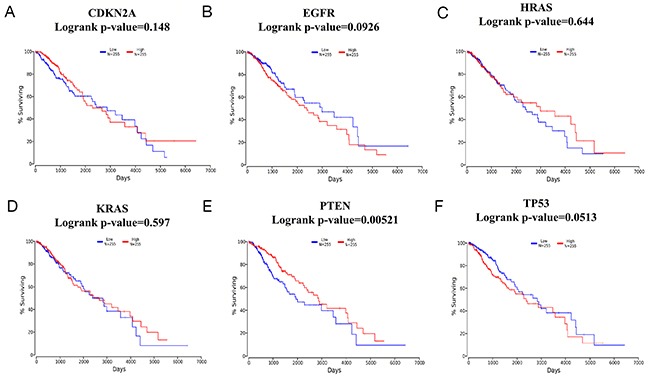
Survival analysis according to CDKN2A/EGFR/HRAS/KRAS/PTEN/TP53 mRNA expression in brain low-grade glioma (LGG) **(A-F)**. A total of 510 LGG samples were included in the OncoLnc database and classified according to the meanvalue of the mRNA expression levels. Blue lines indicate lower levels of mRNAexpression, while red lines indicate higher levels of mRNAexpression.

**Figure 7:**
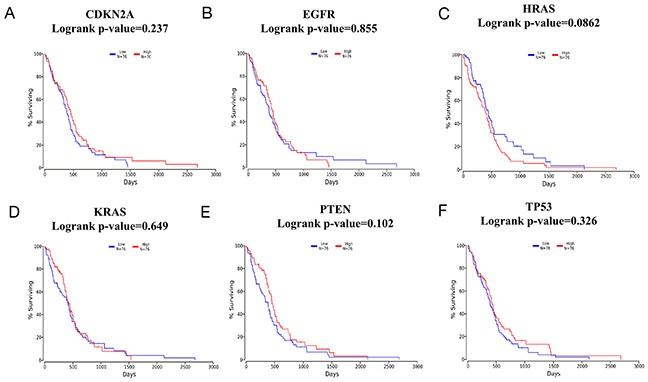
Survival analysis according to CDKN2A/EGFR/HRAS/KRAS/PTEN/TP53 mRNA expression in glioblastoma multiforme (GBM) **(A-F)**. A total of 152 GBM samples were included in the OncoLnc database and classified according to the meanvalue of the mRNA expression level. Blue lines indicate lower mRNA expression levels, while red lines indicate higher mRNA expression levels.

To explore the association between TMZ-related genes and the survival of patients with glioma, OncoLnc, an integrated data mining system, was used. The 6 selected genes (CDKN2A, EGFR, HRAS, KRAS, PTEN and TP53) identified in the glioma pathway were used to conduct a survival analysis with clinical profiles in glioma. The results indicated that in LGG, a high level of PTEN expression predicted longer survival with statistical significance (log-rank *p-value*=0.00521) when patients were classified according to the mean value of the mRNA expression level (Figure 6A-6F). In contrast, in GBM, no gene was statistically significantly associated with patient survival (Figure 7A-7F), which might be attributed to 2 reasons: (1) as shown in Figure 7A-7F, the average survival time for GBM patients was only 500 days, which is consistent with relevant studies; and (2) the OncoLnc analysis was based on a relatively small sample (a total of 152 cases), and the findings could easily be spurious.

## DISCUSSION

Since the landmark findings regarding the antineoplastic activity of TMZ reported in 1984 [4], more than 5529 publications on TMZ have appeared on PubMed. A wide range of biological and cellular activities have been identified for TMZ, demonstrating the fascinating nature of this compound regarding a plethora of related diseases. However, tumor resistance to TMZ is still a critical barrier to the effective treatment of glioma. As such, new analytical means or platforms are required to bridge TMZ with its primary or secondary targets and thereby illustrating the underlying mechanisms of TMZ and its clinical outcomes.

In this study, we performed a functional network analysis using a set of web-based tools. First, we demonstrated the feasibility of analyzing the connectivity between TMZ and cancer using DrugBank, STRING, WebGestalt and ClueGO. As reported in other studies, TMZ acted mainly through 3 DNA repair systems: MRMT, MMR and BER. Based on these 3 DNA repair systems, 5 common genes (MSH2, MSH6, TOP2B, TP53 and XRCC3) were identified using STRING (Figure 1). Notably, TMZ has been reported to induce apoptosis in melanoma cells, and the inactivation of MGMT results in a high level of resistance to TMZ and impairs the expression of MSH2/MSH6 through the over expression of P53 [30]. Furthermore, other studies have shown that the expression levels of TOP2A/Bare significantly higher in human GBM and that TOP2B transcription is corrected in PDGF (+) PTEN (−/−) or PDGF (+) PTEN (−/−) P53 (−/−) models by susceptibility to cancer drugs [20]. For XRCC3, a double-strand break repair gene, the thr241Met polymorphism of XRCC3 has been associated with susceptibility to developing astrocytomas and GBM [31, 32]. Therefore, these findings suggested the hypothesis that TMZ might exert anti-tumor effects through MSH2/MSH6/TOP2B/XRCC3 in glioma patients via a regulated interaction with the TP53 signaling axis.

As supporting evidence, the KEGG enrichment analysis conducted using WebGestalt and ClueGO identified the glioma pathway as significantly altered by TMZ-related genes (Table 4). The association between glioma (GBM and LGG) and the beneficial effects exerted by TMZ in cancer was further observed and evaluated using cBioPortal with6 genes (CDKN2A, EGFR, HRAS, KRAS, PTEN and TP53) identified in the glioma pathway. Both PTEN and TP53 are tumor suppressor genes [33] that participated in almost all the cancer pathways identified by WebGestalt and ClueGO. In the case of GBM, most of the genetic alterations in CDKN2A, PTEN and TP53 were deletions or mutations (Figure 3B), which resulted in a reduction of their expression in conjunction with the development of carcinogenesis [34, 35]. In contrast, for EGFR, most of the genetic alterations were amplifications (Figure 3B), suggesting an over expression during the acceleration of glioma [36, 37]. Moreover, the co-expression analysis illustrated synergistic effects between KRAS and TP53 as well as PTEN and TP53 (Figure 3C), and the mutual exclusivity analysis revealed a tendency toward mutual inhibition between CDKN2A and TP53 and between EGFR and TP53 (Table 6). Thus, the results are consistent with the activation of TP53 by TMZ as a major driver of anti-tumor effects in GBM. Regarding LGG, TP53 (90%) exhibited the most prominent alterations (Figure 5A). Finally, a survival analysis of the 6 genes was conducted in glioma cases (LGG or GBM) using OncoLnc, and the results indicated that high levels of PTEN predicted a statistically significantly longer survival in LGG. This finding is in accordance with the diagnostic significance of PTEN mutation as a molecular marker for poor prognosis in LGG [38, 39]. However, the expression levels of other genes showed no statistically significant associations with survival in either LGG or GBM. Therefore, more large, multicenter clinical trials are urgently required to investigate the association between the expression of TMZ-related genes and provide a molecular biomarker for prognosis in patients with glioma.

In summary, the query of publicly available computational databases may significantly advance research by (1) unraveling the critical role of TMZ in glioma and revealing the mechanisms of glioma. Furthermore, by providing a deeper understanding of TMZ, the current analysis can assist in TMZ bio-curation, and new biological experimental designs will significantly accelerate glioma biology research. (2) This approach should facilitate early disease diagnosis and improve the accuracy of disease prognosis. The candidate genes identified by STRING, WebGestalt and the ClueGO database may facilitate the interpretation of genomic results and thus provide information useful for guiding research. However, several challenges remain for us to investigate and solve: (1) In addition to the glioma pathway, there are other cancer pathways identified by WebGestalt and ClueGO, such as bladder cancer and non-small-cell lung cancer. Therefore, whether the connectivity map shown in this paper to exist between TMZ and glioma can be extended to other solid tumors remains to be investigated. (2) The roles of drug targets detected by STRING must be further explored in the glioma pathway and in other signaling pathways under TMZ control. (3) Genomic alterations in the LGG samples differed from those in the GBM cases; these alterations may play a role in transition from low-grade glioma to high-grade glioma. Therefore, these genomic differences between LGG and GBM can be used to direct future research with reasonable experimental feasibility based on a functional network analysis. Overall, this paper provides a simple yet flexible procedure to test and validate reasonable hypotheses regarding genetic alterations in glioma by applying available biological information, such as in BioGPS, to assist researchers in translating basic studies to clinical applications.

## MATERIALS AND METHODS

### Drug-target search

DrugBank is a web-based bioinformatic database containing comprehensive biochemical and pharma-cological information about drugs, their pharmacological mechanisms and their targets [40, 41]. The tool contains over 4100 drug entries consisting of over 800 FDA-approved small molecules and biotech drugs as well as over 3200 experimental drugs. In this study, DrugBank was used to probe the category and indications for TMZ and the interaction between TMZ and its targets to provide insights regarding the TMZ target network.

### Pathway enrichment analysis and network generation

STRING v10.0 is an online database tool to analyze PPIs for differentially expressed genes and provide interaction information predicted by comparative genomics [42]. In this study, human PPIs for target genes involved in three DNA repair systems were constructed using the STRING database. WebGestalt is a comprehensive web-based integrated data mining system that provides the maximum flexibility for functional enrichment analyses [43]. Biochemical pathways and functions related to the TMZ gene set were specifically queried with a KEGG pathway enrichment analysis in WebGestalt, and the top 15 pathways with an adjusted *p*-value<0.01 were selected. ClueGO is a cytoscape plug in app that visualizes non-redundant biological terms for large clusters of gene sets in a functionally grouped network [44, 45]. In our study, theenrichment analysis of gene-GO terms and bio-pathways was statistically validated with the cytoscape plugin ClueGO + Cluepedia app.

### Cancer genomics data linked to TMZ and glioma survival analysis

The cBioPortal is an open-access resource for interactively exploring multidimensional cancer genomics datasets [46, 47]. OncoLnc is a tool for studying survival correlations and downloading clinical data combined with expression profiles for mRNAs, miRNAs and long non-coding RNAs (lncRNAs) [48]. In this study, the cBioPortal database was used to analyze connectivity of TMZ-related genes across all glioma studies; these genes were then classified as altered or not altered in glioma samples. The altered genes were then further studied using a Kaplan-Meier analysis to evaluate glioma survival according to gene expression using OncoLnc.

## SUPPLEMENTARY MATERIALS TABLE




