# Supplementary material for: Functional network analysis of gene-phenotype connectivity associated with temozolomide
Source: Oncotarget. 2017 Sep 12;8(50):87554–67. doi: 10.18632/oncotarget.20848 (PMC5675653; doi:10.18632/oncotarget.20848)
Supplement: Supplementary file 1 [file oncotarget-08-87554-s001.pdf]

Functional network analysis of gene-phenotype connectivity associated with temozolomide

SUPPLEMENTARY MATERIALS

Supplementary Table 1: Output of search and analysis by STRING on MGMT associated protein-protein interaction (PPI) related to Temozolomide.

See Supplementary File 1
