# Supplementary material for: Functional network analysis of gene-phenotype connectivity associated with temozolomide
Source: Oncotarget. 2017 Sep 12;8(50):87554–67. doi: 10.18632/oncotarget.20848 (PMC5675653; doi:10.18632/oncotarget.20848)
Supplement: Supplementary file 2 [file oncotarget-08-87554-s002.docx]

**Supplemental Table1: Output of search and analysis by STRING on MGMT associated protein-protein interaction (PPI) related to Temozolomide.**

| Searched_Drug (1/1) | | PPI (51) | | |
| --- | --- | --- | --- | --- |
| DB_ID | Name | Target_symbol | PPI_symbol | PPI_ID |
| DB00853 | Temozolomide | MGMT | ACLY | 1844250 |
|  |  |  | ALKBH1 | 1842606 |
|  |  |  | ALKBH2 | 1852650 |
|  |  |  | ALKBH3 | 1848387 |
|  |  |  | APEX1 | 1842615 |
|  |  |  | BIRC5 | 1848314 |
|  |  |  | BRCA1 | 1861387 |
|  |  |  | BRCA2 | 1857163 |
|  |  |  | CDH1 | 1844988 |
|  |  |  | CDH13 | 1860733 |
|  |  |  | CDKN2A | 1859838 |
|  |  |  | CDKN2B | 1846519 |
|  |  |  | DAP | 1843269 |
|  |  |  | DAPK1 | 1853632 |
|  |  |  | DNMT1 | 1853859 |
|  |  |  | EGFR | 1846445 |
|  |  |  | ERCC1 | 1842178 |
|  |  |  | ERCC2 | 1857962 |
|  |  |  | ESR1 | 1842435 |
|  |  |  | FHIT | 1852550 |
|  |  |  | FLT1 | 1852550 |
|  |  |  | GLTSCR1 | 1858418 |
|  |  |  | GSTP1 | 1858630 |
|  |  |  | HIC1 | 1849681 |
|  |  |  | HIF1A | 1852062 |
|  |  |  | HRAS | 1849235 |
|  |  |  | IDH1 | 1844837 |
|  |  |  | IFNB1 | 1857184 |
|  |  |  | KRAS | 1844404 |
|  |  |  | MLH1 | 1843304 |
|  |  |  | MSH2 | 1843341 |
|  |  |  | MSH6 | 1843401 |
|  |  |  | NTHL1 | 1842717 |
|  |  |  | OGG1 | 1848859 |
|  |  |  | PMS1 | 1860608 |
|  |  |  | PMS2 | 1845816 |
|  |  |  | PTEN | 1855586 |
|  |  |  | RARB | 1851458 |
|  |  |  | RASSF1 | 1853458 |
|  |  |  | RUNX3 | 1852701 |
|  |  |  | TIMP3 | 1845855 |
|  |  |  | TOP2A | 1860923 |
|  |  |  | TOP2B | 1859958 |
|  |  |  | TP53 | 1846083 |
|  |  |  | TRIP12 | 1846877 |
|  |  |  | XRCC1 | 1845233 |
|  |  |  | XRCC3 | 1852691 |
|  |  |  | ZBTB33 | 1849688 |
|  |  |  | ZBTB38 | 1860635 |
|  |  |  | ZBTB4 | 1849010 |
